# Additional Erythrocyte Field Is Helpful for Graphic Type Differentiation of Cell Count Determination Between Acute Periprosthetic Joint Infection and Hematoma

**DOI:** 10.3390/antibiotics15020122

**Published:** 2026-01-26

**Authors:** Florian Hubert Sax, Marius Hoyka, Benedikt Paul Blersch, Elke Weissbarth, Philipp Schuster, Irina Berger, Hansjörg Baum, Bernd Fink

**Affiliations:** 1 Department of Joint Replacement, General and Rheumatic Orthopaedics, Orthopaedic Clinic Markgröningen gGmbH, Kurt-Lindemann-Weg 10, 71706 Markgröningen, Germany; florian.sax@rkh-gesundheit.de (F.H.S.); marius.hoyka@rkh-gesundheit.de (M.H.); benedikt.blersch@rkh-gesundheit.de (B.P.B.); philipp.schuster@rkh-gesundheit.de (P.S.); 2 Department of Trauma and Reconstructive Surgery, BG Klinik, University of Tübingen, Schnarrenbergstraße 95, 72076 Tübingen, Germany; 3 Institute for Laboratory Medicine, RKH Klinikum Ludwigsburg, Location Orthopaedic Clinic Markgröningen, Kurt-Lindemann-Weg 10, 71706 Markgröningen, Germany; elke.weissbarth@rkh-gesundheit.de (E.W.); hansjoerg.baum@rkh-gesundheit.de (H.B.); 4 Department of Orthopedics and Traumatology, Clinic Nuremberg, Paracelsus Medical Private University, Breslauer Straße 201, 90471 Nürnberg, Germany; 5 Institute of Pathology, Klinikum Kassel, Mönchebergstraße 41-43, 34125 Kassel, Germany; irina.berger@gnh.net; 6 Orthopaedic Department, University Hospital Hamburg-Eppendorf, Martinistrasse 52, 20246 Hamburg, Germany

**Keywords:** periprosthetic joint infection, hematoma, diagnosis, leukocyte, cell count, aspiration

## Abstract

**Background/Objectives**: This study was designed to verify the hypothesis that graphical cell differentiation of synovial cell count analysis is helpful for diagnosis of acute periprosthetic joint infection (PJI) and that the additional erythrocyte field has advantages to differentiate PJI from hematoma. **Methods**: A total of 77 total knee arthroplasties and 31 total hip arthroplasties underwent aspiration within six weeks of primary implantation. The aspirate was analyzed with the cell counter Yumizen H500 and examined by cultivation. Serum CRP was also determined. A total of 43 patients underwent revision and microbiological and histological analysis of the periprosthetic tissue, according to Morowitz and Krenn, was performed. The ICM criteria for diagnosing PJI were used. **Results**: Thirty-two cases (29.6%) were classified as acute infection. The graphical type differentiation LMNE (leukocyte–monocyte–neutrophil–eosinophil) showed 28 cases with type II (infection type), 63 cases with type IV (indifferent type), 13 cases with type V (hematoma type with a peak in the erythrocyte field) and 4 cases with type VI (mixed infection and hematoma). The LMNE matrix assessment had an accuracy of 98.7%, sensitivity of 96.9%, specificity of 98.7%, positive predictive value of 96.9%, negative predictive value of 98.7%, a positive likelihood ratio of 73.62, and a negative likelihood ratio of 0.03. Only one single non-infectious hematoma sample was misclassified as type VI (mixed infection/hematoma). **Conclusions**: The graphical type differentiation of the cell count analysis of synovial aspirates is a helpful method for diagnosis of acute periprosthetic joint infection and differentiating between hematoma and real early periprosthetic infections. This report shows that the new erythrocyte field of the Yumizen H500 is a useful additional diagnostic tool.

## 1. Introduction

With the growing number of prosthetic interventions, the absolute incidence of otherwise rare complications is rising, rendering them an increasingly significant concern in routine clinical practice. Acute periprosthetic joint infection (PJI) is a rare but severe complication following joint arthroplasty, with reported incidence rates for total hip and knee replacements ranging from 0.16% to 0.6% [1,2]. Early postoperative diagnosis is often challenging, as physiological wound healing responses, such as swelling, erythema, and warmth, can mimic the initial clinical signs of infection [1,2,3]. Timely and accurate differentiation is crucial, as delayed intervention may permit the development of a mature biofilm on the implant surface within approximately 3–4 weeks [4]. Once established, such biofilms substantially increase bacterial resistance to antimicrobial therapy and host immune defenses, thereby complicating eradication and often necessitating more invasive revision strategies.

One of the most established diagnostic tools for detecting PJI is the determination of the synovial fluid leukocyte count (WBC) [1,2,3,5], and it constitutes a core element in both the Musculoskeletal Infection Society (MSIS) and the more recent International Consensus Meeting (ICM) criteria [6,7,8,9]. Reported cut-off values for acute PJI with high sensitivity vary between studies: 10,700 cells/µL for total knee arthroplasties in Bedair et al. [1], 11,200 cells/µL in Kim et al. [2], 12,800 cells/µL for total hip arthroplasties in Yi et al. [3], as well as 8910 cells/µL in Yu et al. [5] and 10,170 cells/µL in Sukhonthamarn et al. [10] for both hip and knee replacements. The consensus meeting established a threshold of 10,000 cells/µL combined with ≥90% polymorphonuclear leukocytes (PMN) in the aspirate and a serum CRP of 100 mg/L for the diagnosis of acute PJI [9]. However, these WBC levels overlap with those found in peripheral blood, meaning that postoperative intraarticular hematomas may present with similar leukocyte counts [1,3].

The percentage of PMN in the synovial aspirate is another important diagnostic parameter [6,7,8,9], with reported cut-offs of 89% in Bedair et al. [1], Kim et al. [2] and Yi et al. [3], but 79.5% in Sukhonthamarn et al. [10]. But also, PMN percentages can be influenced by postoperative hematomas, further complicating the distinction between acute infection and sterile postoperative hematomas.

To address these diagnostic limitations within the early postoperative phase, our group has investigated automated synovial fluid cell count analysis as a rapid adjunctive tool [11,12,13]. In our previous work using the ABX Pentra XL 80 cell counter (Horiba Medical, Montpellier, France), LMNE plots that map cellular and particulate components according to their optical characteristics were generated. This approach exploits the distinct light absorption properties of different cells and prosthetic wear particles, enabling differentiation between acute PJI and early postoperative hemarthrosis without infection [11,12,13].

Based on this methodology, we identified six characteristic LMNE patterns: type I (abrasion), type II (infection), type III (mixed infection/abrasion), type IV (indifference), type V (hemarthrosis), and type VI (mixed hemarthrosis/infection) [11,12,13]. Classification into infection-associated (types II and VI) versus non-infectious (types IV and V) patterns yielded a sensitivity of 100%, a specificity of 97.3%, and a positive likelihood ratio of 37.0 for diagnosing early PJI [12].

**Figure 1:**
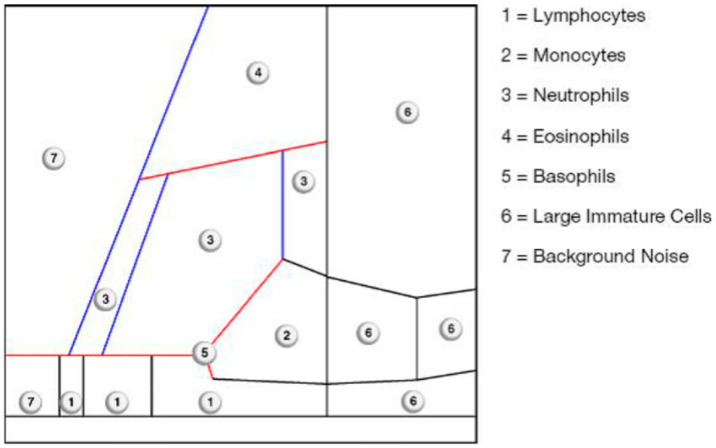
LMNE matrix with the different areas corresponding to the leukocyte populations and the NOISE area of the Yumizen H500.

**Figure 2:**
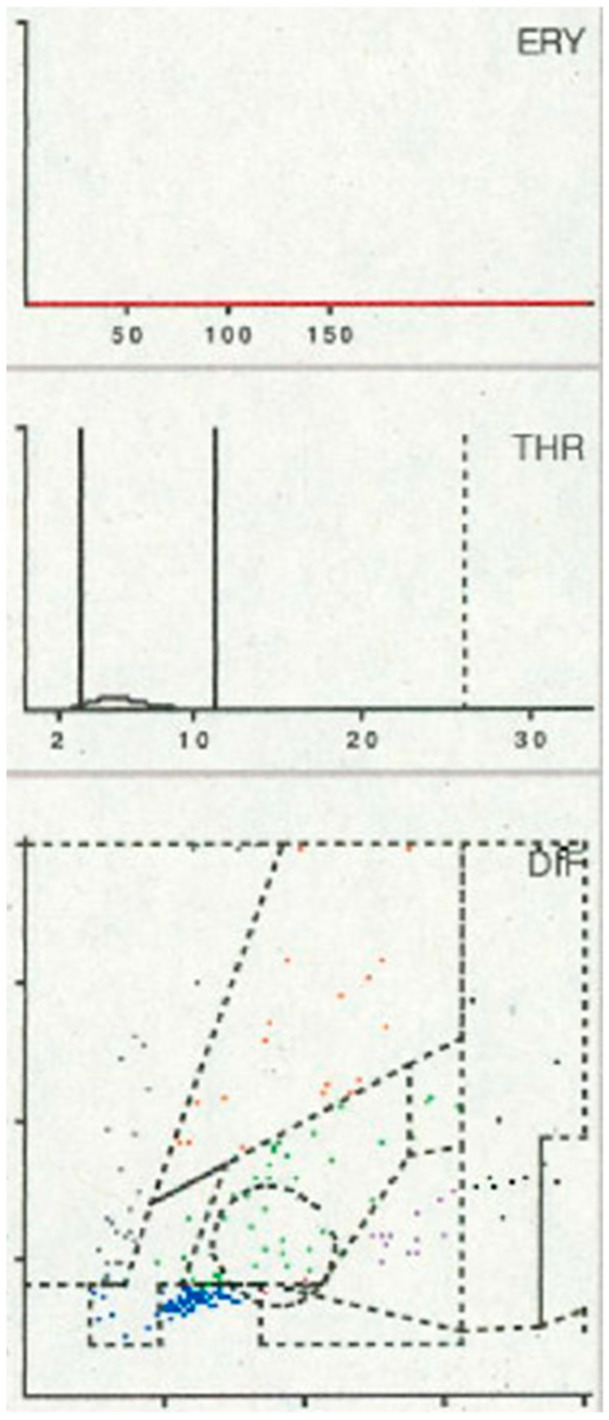
Type IV LMNE matrix (indifference type) with no clusters and no peak in the erythrocyte field in an 87-year-old female patient 6 weeks after total knee arthroplasty with effusion. The laboratory assessment showed a CRP of 125 mg/L and a peripheral blood leukocyte count of 13,200/µL, while synovial fluid analysis revealed 370 cells/µL, of which 30.2% were polymorphnuclear cells. Microbiological testing remained sterile.

**Figure 3:**
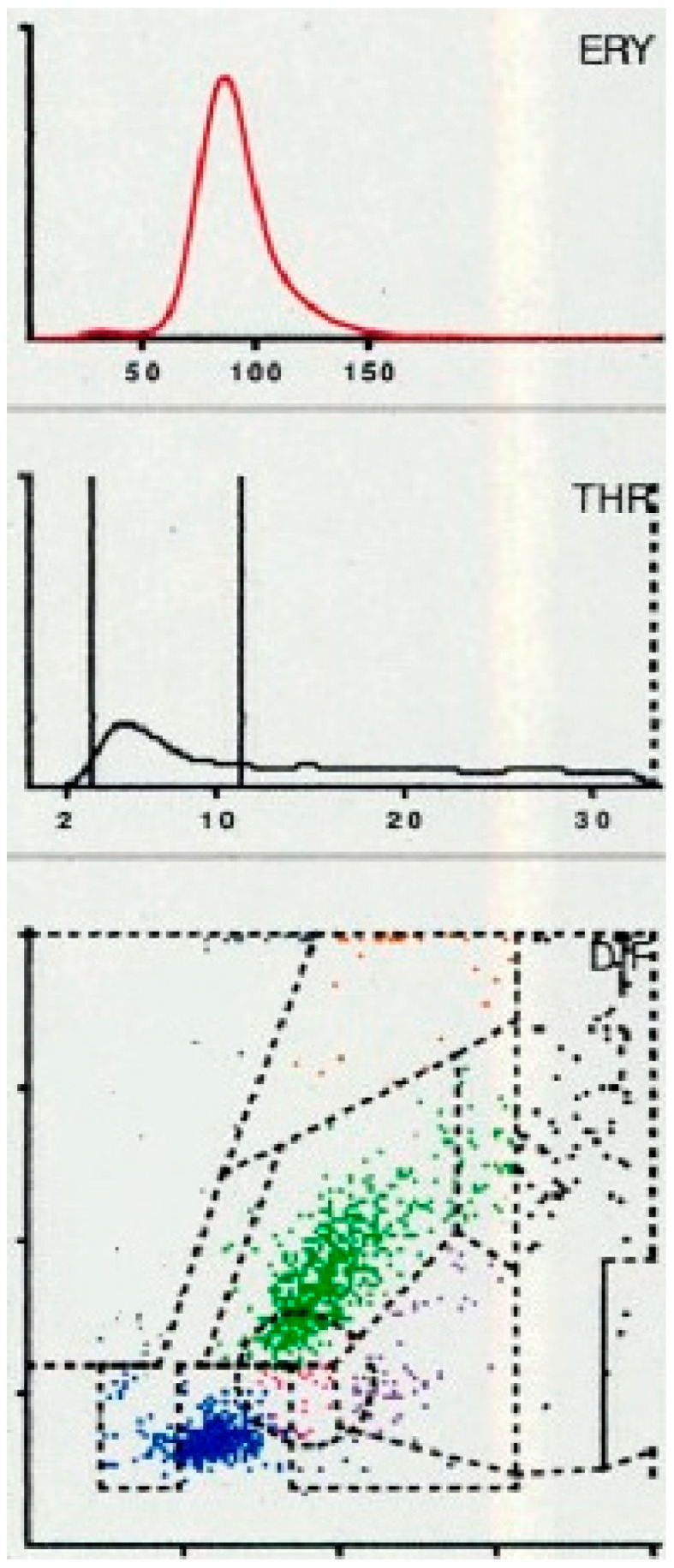
Type V LMNE matrix (hematoma type) with a cloud in the field of the lymphocytes and another clear cloud in the field of the neutrophils as well as a clear peak in the erythrocyte curve in a 74-year-old female patient 3 weeks after total knee arthroplasty. The laboratory assessment showed a CRP of 78 mg/L and a peripheral blood leukocyte count of 10,100/µL, while synovial fluid analysis revealed 6400 cells/µL, of which 48.4% were polymorphnuclear cells. Microbiological testing remained sterile.

**Figure 4:**
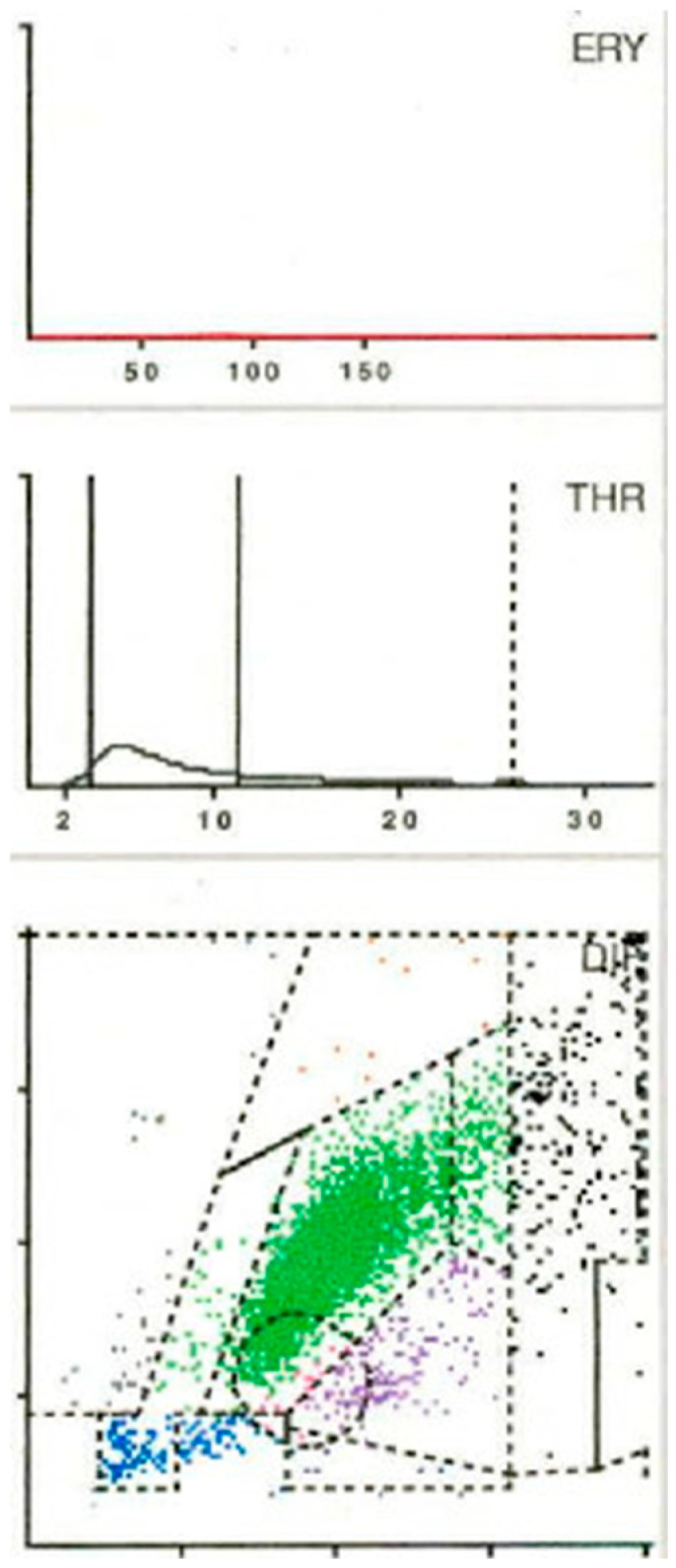
Type II LMNE matrix (infection type) with a cloud in the area of the neutrophils and a flat curve in the erythrocyte field in a 67-year-old male patient 4 weeks after total knee arthroplasty. The laboratory assessment showed a CRP of 75.4 mg/L and a peripheral blood leukocyte count of 11,400/µL, while synovial fluid analysis revealed 16,600 cells/µL, of which 90.4% were polymorphnuclear cells, and microbiological testing identified *Staphylococcus epidermidis*.

With the introduction of the Yumizen H500 (Horiba, Montepellier, France) as the successor to the ABX Pentra XL 80 (Horiba, Montepellier, France), LMNE plots now exhibit slightly altered graphical features and include additional analytical fields for detecting blood admixture in synovial aspirates (Figure 1, Figure 2, Figure 3 and Figure 4). Preliminary experience suggests that LMNE-type differentiation is equally feasible with the new device; however, its diagnostic accuracy for distinguishing acute PJI from postoperative hemarthrosis remains to be systematically evaluated as well as the value of the new additional erythrocyte field [13].

The aim of this study was therefore to test the following hypotheses:

**H1.**  *Graphic type differentiation helps in the diagnosis of acute periprosthetic infection and also allows differentiation from hematoma with the Yumizen H500.*

**H2.**  *The diagnostic value of the graphic cell count differentiation is at least as high with the Yumizen H500 as with the predecessor model Pentra XL 80.*

**H3.**  *The additional, new erythrocyte field supports the identification of the proportion of blood admixtures in the aspirate and facilitates the differentiation between acute infection and hematoma.*

## 2. Results

Out of the total cohort, 32 cases (29.6%) met the International Consensus Meeting (ICM) criteria for infection. The mean cell count in the aspirate of cases with infection was 49,124 cells/µL (1720–211,060 cells/µL) and thus significantly higher than that of cases without infection at 1964/µL (170–14,490/µL) (*p* < 0.001). However, five cases without infection had a higher cell count than the limit of 10,000 cells/mL set by the ICM [6,7,8,9].

The proportion of polymorphonuclear leukocytes (PMNs) was likewise markedly increased in infected individuals—77.3% (23.1–93.8%)—compared with non-infected individuals, who showed 48.6% (13.6–97.2%) (*p* < 0.001). Depending on the threshold applied, the resulting diagnostic distributions and accuracies are provided in Table 1. Combining cell count and PMN percentage improved specificity at the expense of sensitivity (Table 1).

Serum C-reactive protein (CRP) concentrations were also significantly elevated in the infection group, with median levels of 97.6 (10–334.1) mg/L, compared with 38.1 (1.4–86.7) mg/L in the non-infection group (*p* < 0.001). Here, eight patients without infection had a CRP value higher than 95 mg/L, which was defined as the threshold for early periprosthetic failure in the studies by Bedair et al. [1] and Yi et al. [3].

**Figure 5:**
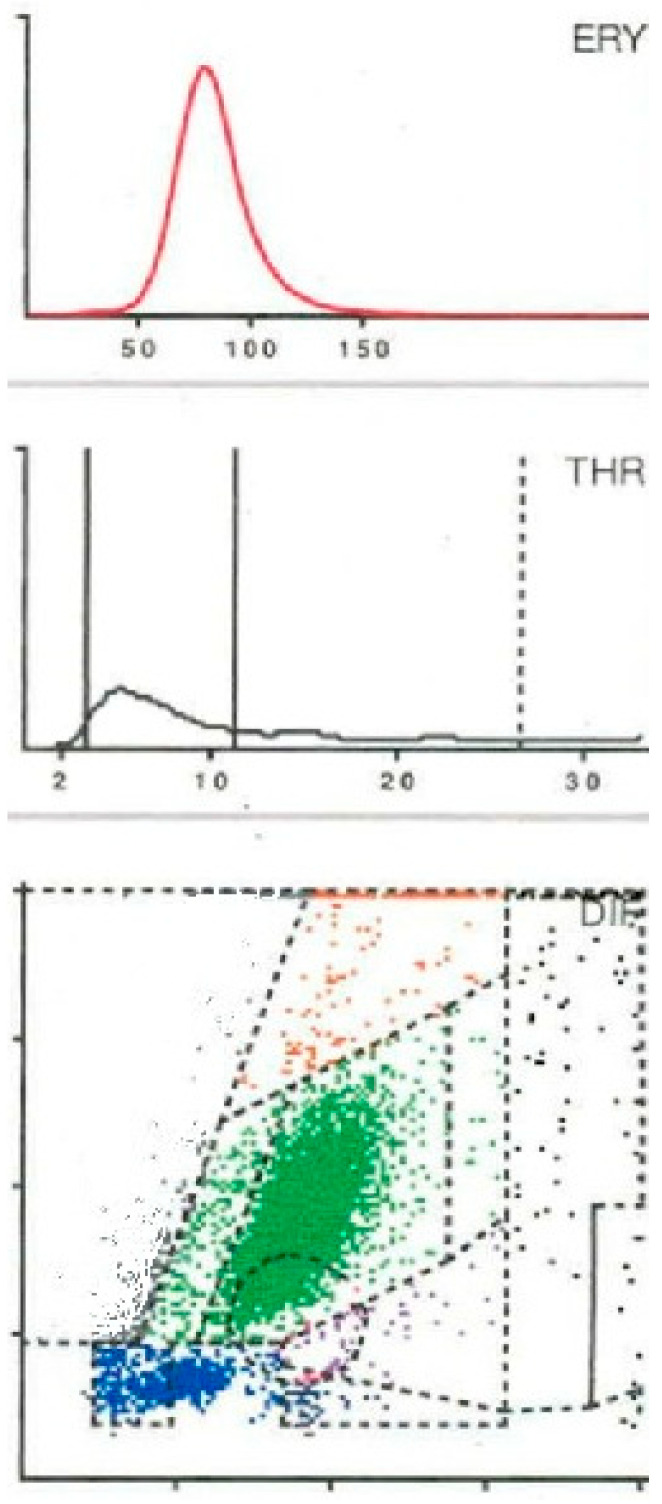
Type VI LMNE matrix (combined type of infection and hematoma) in a 90-year-old female patient 5 weeks after total knee arthroplasty. The laboratory assessment showed a CRP of 116 mg/L and a peripheral blood leukocyte count of 12,200/µL, while synovial fluid analysis revealed 14,800 cells/µL, of which 89.4% were polymorphnuclear cells. Microbiological testing identified *Staphylococcus caprae*.

When LMNE (leukocyte–monocyte–neutrophil–eosinophil) pattern matrices were analyzed, type IV (indeterminate) morphology (Figure 2) was detected in 63 cases and type V (hematoma type) (Figure 3) in 13 cases; both were classified as non-infectious patterns. Type II (infection type) (Figure 4) occurred in 28 cases, while type VI (mixed infection/hematoma type) (Figure 5) was identified in four cases, yielding a total of 32 LMNE analyses consistent with infection (Table 1). At 54,082 cells/µL (1720 to 211,060 cells/µL), the median for joints with LMNE type II (infection type) was significantly higher than for joints with LMNE type V (hematoma) (median 7059 cells/µL, from 220–14,490 cells/µL) (*p* = 0.006). A total of 5 of the 13 joints with type V had a cell count higher than the cut-off value of 10,000 cells/µL and 11 of the 28 joints with type II had a cell count below this cut-off value. The percentage of polymorphonuclear leukocytes (PMN) was not significantly different in these two LMNE groups (type II: median 76.7%, from 33.2 to 93.8%; type V: median 72.2%, from 43.8 to 97.2%; *p* = 0.356).

Overall, LMNE matrix assessment demonstrated excellent diagnostic performance, with an accuracy of 98.7%, sensitivity of 96.9%, specificity of 98.7%, positive predictive value of 96.9%, negative predictive value of 98.7%, a positive likelihood ratio of 73.62, and a negative likelihood ratio of 0.03. Only one single non-infectious hematoma sample was misclassified as type VI (mixed infection/hematoma) (Table 1).

Microbiological culture of the aspirates yielded a sensitivity of 68.8% and specificity of 100% as shown in Table 1. No statistically significant differences were observed between sexes for any of the diagnostic parameters.

## 3. Discussion

The study shows that graphic LMNE visualization was the only preoperative test with high sensitivity and specificity. Both the simple cell count with a cut-off of over 10,000 and the percentage of polymorphonuclear leukocytes in the aspirate showed good specificities but insufficient sensitivities, so patients with infections could have been overlooked. The preoperative CRP value was also unable to demonstrate good sensitivity at any of the various cut-off values. Thus, the graphical representation with the Yumizen H500 shows the best accuracy and value in the diagnostic differentiation between an early infection and a hematoma and therefore for the diagnosis of an early periprosthetic infection.

The results reinforce those of the first publication with the predecessor model Pentra XL 80 by Fink et al. [12]. The specificity of the graphical representation was even slightly higher in the present study with the Yumizen H500 at 98.7% compared to 97.3% with the Pentra XL 80, and the likelihood ratio of 73.62 with the Yumizen H500 was higher compared to 37.0 with the Pentra XL 80. The positive likelihood ratio indicates the factor by which a positive result is more likely to occur in diseased individuals than in healthy individuals; thus, for the Yumizen H500, it is again approximately twofold higher compared with the Pentra XL 80. This may be due to the fact that the additional red blood cell field in the Yumizen H500 makes it easier to identify hematomas as such, and, therefore, the quantitative proportion of the hematoma in the aspirate can be better estimated. This makes it easier to estimate the extent to which the measured leucocytes are caused by a haematoma or blood admixture and not by a possible infection, so that the measured leucocyte count can then be “corrected” downwards, as it were.

The simple cell count in the aspirate with a threshold value of 10,000 cells/microlitre had a good specificity of 93.4%, so few false-positive interpretations are to be expected, but with a sensitivity of only 59.4% a high number of false-negative interpretations must be expected. The same applies to the number of polymorphonuclear leucocytes in the aspirate with a specificity of 95.7% at a cut-off of 79.5% PMN and even 98.6% at a cut-off of 90% PMN, but a sensitivity of 56.3% at the first cut-off and only 15.6% at the second cut-off. The combination of both parameters even improved the specificity to 100% but not the sensitivity (Table 1). This means that these tests have a high diagnostic value when the result is positive, indicating the presence of an infection, but a poor diagnostic value when the result is negative, indicating the absence of an infection. Therefore, they are not suitable as standalone tests to rule out early postoperative periprosthetic infections.

In the present study, serum CRP showed the lowest values among the evaluated markers, particularly with regard to sensitivity and thus its ability to exclude early postoperative infections. This observation may be attributed to the typical postoperative kinetics of CRP, which generally normalizes within 2–4 weeks but can remain elevated for up to 60 days without the presence of infection [14,15,16,17]. Moreover, serum CRP levels reflect systemic inflammatory activity with an approximate delay of 60 h relative to local tissue events; consequently, early periprosthetic or joint infections may already be evolving while serum CRP concentrations still remain below the diagnostic cutoff [14,15,16,17]. Furthermore, the cut-off value for serological CRP for the diagnosis of acute PJI is also unclear. Values in a wide range, between 24 and 95 mg/L, are reported in the literature [2,17]. However, the choice of the lower cut-off of 75 mg/L in our study only increased the sensitivity to 50% (from 40.6% for CRP > 100 mg/L). Therefore, the cut-off values for the previous preoperative tests (serum CRP, synovial cell count and synovial fraction of PMN) remain unclear and are probably dependent on numerous influencing factors (including time after surgery and symptom onset, type of microorganism and immune status of the patient). Therefore, it appears that the cut-off values chosen in the ICM for these parameters for acute PJI are not supported by substantial evidence in the literature.

Thus, the additional use of the graphical LMNE type determination seems to provide information that facilitates the diagnosis of acute periprosthetic infections, whereby the new erythrocyte curve in the Yumizen H500 seems to have a supportive effect. This method is especially useful for differentiating infectious processes from postoperative hematoma, which is clinically relevant since postoperative edema and swelling often make this distinction challenging. Furthermore, the cell count in cases of hemarthrosis may reach or even approximate the diagnostic threshold for infection as defined by the International Consensus Meeting (ICM) and other authors [1,2,3,4,5,6,7,8,9,10].

The study has strengths and weaknesses. The strength of this study lies in the standardized implementation of the procedure. One limitation of the study is that an infection was only confirmed in around 40% of cases via the results of a revision surgery using several diagnostic criteria (cultivation of the aspirate and five tissue samples, histology and CRP determination). In the other patients, the usual preoperative parameters were used for verification using the 2018 ICM criteria [7]. This is due to the fact that not every suspected periprosthetic infection automatically leads to a revision, but the preoperative diagnosis serves to identify an indication for revision. However, other non-infectious indications (such as hematoma removal and dislocation of a total hip arthroplasty) were also included. In addition, the total number of cases was relatively small. This is due to the low rate of early periprosthetic infections and the need for revisions within the first 6 weeks after implantation of a knee or hip prosthesis. However, the number of cases was sufficient to demonstrate the positive effect of the graphical representation of aspirates for the differentiation of hematomas and infections. Despite these findings, the results of the current study and the proposed classification scheme should be confirmed in larger patient populations to ensure reproducibility. Similarly to histopathological assessments, the categorization of cell patterns is partly influenced by the examiner’s subjective interpretation and level of experience. Although observer agreement was high in our study and the use of the Yumizen H500 facilitated visual analysis, interpretation of LMNE matrices inevitably retains an element of subjectivity. It must also be acknowledged that LMNE matrix generation is not universally available, as it requires optical assessment of light absorption, an analysis feature lacking in many routine cell counters that rely solely on scattered light and particle dimension.

## 4. Materials and Methods

Between 2021 and 2025, patients with aspiration within six weeks of a primary arthroplasty were prospectively enrolled. A total of 108 cases met the initial inclusion criteria, comprising 77 total knee arthroplasties and 31 total hip arthroplasties; the cohort consisted of 53 women and 55 men. The mean age of the analyzed cohort was 70.9 ± 10.6 years (range, 46–90 years). All patients underwent aspiration of the joint. The interval from primary implantation to aspiration averaged 3.9 ± 2.1 weeks (range, 1–6 weeks). In the majority of cases (*n* = 63), aspiration was prompted by clinical suspicion of an early infection or an infected hematoma, whereas effusion of the knee (*n* = 33) and instability with dislocation accounted for 12 hip revisions.

In all cases, joint aspiration was carried out. On one hand, the aspirated fluid was immediately transferred into pediatric blood culture bottles containing BD BACTEC-PEDS-PLUS/F Medium (Becton Dickinson, Heidelberg, Germany) and incubated for 14 days [18]. On the other hand, a cell count was determined from the aspirate. For this purpose, at least 1 mL synovia was placed in an EDTA tube for determining the cell count with the laboratory diagnostic device Yumizen H500 (Horiba Medical, Montpellier, France). The Yumizen H500 is a laboratory diagnostic device for the analysis of the cell count and the WBC-differentiation of blood and also of body fluids. Here, we selected the so-called 5-DIFF mode from the various processing modes available. A total of 26 laboratory parameters are recorded, including the 5 cell types, eosinophils, neutrophils, monocytes, lymphocytes and basophils, as well as atypical lymphocytes and large, immature cells. These cell types are graphically mapped in a so-called LMNE matrix depending on their cell volume (x-axis) and their light scattering or refraction and absorption (y-axis) (Figure 1). The analysis is based on an impedance measurement, flow cytometry and cytochemistry. The volume difference of the cells due to the impedance measurement is shown graphically on the X-axis and the differentiation of the light absorption in flow cytometry on the Y-axis of the LMNE matrix. This enables the graphical assignment and thus differentiation of the 4 leukocyte populations: lymphocytes, monocytes, neutrophils and eosinophils (Figure 1). The “NOISE” region in the LMNE matrix typically reflects non-specific signals or cellular debris. The graphical classification of leukocyte populations using the LMNE matrix represents a widely recognized approach in hematological diagnostics [19,20,21]. In all cases, serum C-reactive protein (CRP) levels were measured.

The evaluations and assignment of the matrices to the different types were carried out twice by two examiners (FHS and BF), independently of one another and without knowledge of the histology. It showed high reliability, with an intrarater intraclass correlation coefficient of 0.99 and of 0.98 between raters, respectively.

A total of 6 LMNE types have been defined in previous studies [11,12,13] (type I = abrasion type, type II = infection type (Figure 4), type III = mixed type with abrasion and periprosthetic infection, type IV = indifferent type, type V = hematoma type (with smaller plots in the fields for the eosinophils, monocytes, lymphocytes and neutrophils and a peak in the erythrocyte curve) (Figure 3) and type VI as mixed type II between infection (with a strong plot in the neutrophil field and hematoma with the corresponding smaller plots for the other white blood cells and a peak in the erythrocyte curve) (Figure 5)).

To determine the presence or absence of a periprosthetic joint infection (PJI), all diagnostic steps were performed in accordance with the International Consensus Meeting (ICM) criteria [6,7,8,9]. A PJI was considered confirmed when the cumulative diagnostic score reached at least six points.

A total of 43 cases underwent revision surgery. During the surgical procedure, five separate samples were obtained from different locations in close proximity to the prosthesis (synovium and periprosthetic tissue). An additional five samples from the synovium and the periprosthetic connective tissue membrane were collected for histopathological examination. Perioperative antibiotic administration was strictly withheld until all samples had been collected. All biopsy specimens were placed in sterile containers and, together with the aspirated fluid, transported to the microbiology laboratory within one hour of collection. There, the samples were inoculated onto blood agar plates and into special nutrient broth for anaerobic organisms, followed by incubation for 14 days [18]. Histopathological evaluation included quantification of polymorphonuclear leukocytes per high-power field, as well as classification according to Krenn et al. [22,23] and Morawietz et al. [24]. This system differentiates between type I (debris-associated), type II (infection-associated), type III (mixed type), and type IV (indeterminate).

Statistical analyses were carried out using SPSS software for Windows (version 22.0; IBM Corp., Armonk, NY, USA). For statistical evaluation of parametric data, Student’s *t*-test, and for nonparametric data, a Mann–Whitney U-test was used. All reported *p*-values are two-tailed, with an alpha level < 0.05 considered as significant. All values are either given as median and interquartile range in the result section and as mean and standard deviation in the Materials and Methods section. Diagnostic test performance was evaluated by calculating sensitivity, specificity, and likelihood ratios. All participants provided written informed consent prior to inclusion. The study was conducted in accordance with the ethical principles of the Declaration of Helsinki and was approved by the Ethics Committee of the State Chamber of Physicians of Baden-Württemberg (approval number F-2014-027).

## 5. Conclusions

The graphical evaluation of synovial aspirates using LMNE matrices represents a meaningful addition to the diagnostic work-up of prosthetic joints in the diagnosis of acute periprosthetic joint infection. Of particular value is the erythrocyte field, which provides an additional diagnostic layer by allowing a more reliable distinction between early periprosthetic infection and hemarthrosis-related cell count elevations. This visualization enhances both interpretability and diagnostic precision. Incorporating the graphical cell type differentiation with erythrocyte component into the standard diagnostic algorithm for early periprosthetic joint infection could therefore strengthen clinical decision making and improve diagnostic accuracy in complex postoperative scenarios.

## Figures and Tables

**Table 1:** Diagnostic value of the different diagnostic tests.

Likelihood Ratio Negative	Likelihood Ratio Positive							
LHR neg.	LHR pos.	98.1%	Accuracy		Infection			
0.03	73.62	96.9%	Sensitivity		no	yes		
		98.7%	Specificity	32	1	31	pos.	LMNE type 2/6
		96.9%	PPW	76	75	1	neg.	
		98.7%	NPW	**108**	76	32		
LHR neg.	LHR pos.	75.0%	Accuracy		Infection			
0.66	3.86	40.6%	Sensitivity		no	yes		
		89.5%	Specificity	21	8	13	pos.	CRP > 100 mg/dL
		61.9%	PPW	87	68	19	neg.	
		78.2%	NPW	**108**	76	32		
LHR neg.	LHR pos.	75.9%	Accuracy		Infection			
0.63	4.16	43.8%	Sensitivity		no	yes		
		89.5%	Specificity	22	8	14	pos.	CRP > 90 mg/dL
		63.6%	PPW	86	68	18	neg.	
		79.1%	NPW	**108**	76	32		
LHR neg.	LHR pos.	75.0%	Accuracy		Infection			
0.58	3.45	50.0%	Sensitivity		no	yes		
		85.5%	Specificity	27	11	16	pos.	CRP > 75 mg/dL
		59.3%	PPW	81	65	16	neg.	
		80.2%	NPW	**108**	76	32		
LHR neg.	LHR pos.	83.3%	Accuracy		Infection			
0.43	9.03	59.4%	Sensitivity		no	yes		
		93.4%	Specificity	24	5	19	pos.	Cell Count ≥ 10,000
		79.2%	PPW	84	71	13	neg.	
		84.5%	NPW	**108**	76	32		
LHR neg.	LHR pos.	88.0%	Accuracy		Infection			
0.41	#DIV/0!	59.4%	Sensitivity		no	yes		
		100.0%	Specificity	19	0	19	pos.	Cell Count ≥ 10,000
		100.0%	PPW	89	76	13	neg.	+LMNE pos.
		85.4%	NPW	**108**	76	32		
LHR neg.	LHR pos.	83.3%	Accuracy		Infection			
0.46	13.13	56.3%	Sensitivity		no	yes		
		95.7%	Specificity	21	3	18	pos.	PMN ≥ 79.5%
		85.7%	PPW	81	67	14	neg.	
		82.7%	NPW	**102**	70	32		
LHR neg.	LHR pos.	72.5%	Accuracy		Infection			
0.86	10.94	15.6%	Sensitivity		no	yes		
		98.6%	Specificity	6	1	5	pos.	PMN ≥ 90%
		83.3%	PPW	96	69	27	neg.	
		71.9%	NPW	**102**	70	32		
LHR neg.	LHR pos.	86.3%	Accuracy		Infection			
0.44	#DIV/0!	56.3%	Sensitivity		no	yes		
		100.0%	Specificity	18	0	18	pos.	PMN ≥ 79.5%
		100.0%	PPW	84	70	14	neg.	+Cell Count ≥ 10,000
		83.3%	NPW	**102**	70	32		
LHR neg.	LHR pos.	73.5%	Accuracy		Infection			
0.84	#DIV/0!	15.6%	Sensitivity		no	yes		
		100.0%	Specificity	5	0	5	pos.	PMN ≥ 90%
		100.0%	PPW	97	70	27	neg.	+Cell Count ≥ 10,000
		72.2%	NPW	**102**	70	32		
LHR neg.	LHR pos.	90.7%	Accuracy		Infection			
0.31	#DIV/0!	68.8%	Sensitivity		no	yes		
		100.0%	Specificity	22	0	22	pos.	Culture
		100.0%	PPW	86	76	10	neg.	
		88.4%	NPW	**108**	76	32		
LHR neg.	LHR pos.	93.0%	Accuracy		Infection			
0.09	18.26	91.3%	Sensitivity		no	yes		
		95.0%	Specificity	22	1	21	pos.	Histo > five cells
		95.5%	PPW	21	19	2	neg.	
		90.5%	NPW	**43**	20	23		
LHR neg.	LHR pos.	95.3%	Accuracy		Infection			
0.05	19.13	95.7%	Sensitivity		no	yes		
		95.0%	Specificity	23	1	22	pos.	Histo type 2
		95.7%	PPW	20	19	1	neg.	
		95.0%	NPW	**43**	20	23		

## Data Availability

We do not wish to share our data because some of the patient’s data relate to individual privacy and, according to the policy of our hospital, these data may not be shared with others without permission.

## References

[1] Bedair H., Ting N., Jacovides C., Saxena A., Moric M., Parvizi J., Della Valle C.J. (2011). Diagnosis of early postoperative TKA infection using synovial fluid analysis. Clin. Orthop. Relat. Res..

[2] Kim S.G., Kim J.G., Jang K.M., Han S.B., Lim H.C., Bae J.H. (2017). Diagnostic value of synovial white blood cell count and serum C-reactive protein for acute periprosthetic joint infection after knee arthroplasty. J. Arthroplast..

[3] Yi P.H., Cross M.B., Moric M., Sporer S.M., Berger R.A., Della Valle C.J. (2014). Diagnosis of infection in the early postoperative period after total hip arthroplasty. Clin. Orthop. Relat. Res..

[4] Tornero E., Soriano A. (2016). Importance of Selection and Duration of Antibiotic Regimen in Prosthetic Joint Infections Treated with Debridement and Implant Retention—Authors’ Response. J. Antimicrob. Chemother..

[5] Yu B.Z., Fu J., Chei W., Hao L.B., Chen J.Y. (2020). Neutrophil to lymphocyte ratio as a predictor for diagnosis of early periprosthetic joint infection. BMC Muscoloskeltal. Disord..

[6] Parvizi J., Zmistowski B., Berbari E.F., Bauer T.W., Springer B.D., Della Valle C.J., Garvin K.L., Mont M.A., Wongworawat M.D., Zalavras C.G. (2011). New definition for periprosthetc joint infection: From the Workgroup of the Musculoskeletal Infection Society. Clin. Orthop. Relat. Res..

[7] Parvizi J., Tan T.L., Goswami K., Higuera C., Della Valle C., Chen A.F., Shohat N. (2018). The 2018 Definition of Periprosthetic Hip and Knee Infection: An Evidence-Based and Validated Criteria. J. Arthroplast..

[8] (2011). Workgroup Convened by the Musculoskeletal Infection Society. New definition for periprosthetic joint infection. J. Arthroplast..

[9] Parvizi J., Gehrke T. (2014). International Consensus Group on Periprosthetic Joint Infection. Definition of periprosthetic joint infection. J. Arthroplast..

[10] Sukhonthamarn K., Tan T.I., Xu C., Ku F.C., Lee M.S., Citak M., Gehrke T., Goswani K., Parvizi J. (2020). Determining diagnostic thresholds for acute postoperative periprosthetic joint infection. J. Bone. Joint. Surg. Am..

[11] Fink B., Hoyka M., Weissbarth E., Schuster P., Berger I. (2021). The graphical representation of cell count representation. A new procedure for the diagnosis of periprosthetic joint infections. Antibiotics.

[12] Fink B., Hoyka M., Weissbarth E., Schuster P., Berger I. (2022). A new graphic type differentiation for distinguishing acute periprosthetic joint infection from hemarthrosis. Antibiotics.

[13] Fink B., Hoyka M., Blersch B.P., Baum H., Sax F.H. (2023). Graphic type differentiation of cell count data for diagnosis of early and late periprosthetic joint infection: A new method. Technol. Health Care.

[14] Sigmund I.K., Puchner S.E., Windhager R. (2021). Serum inflammatory biomarkers in the diagnosis of periprosthetic joint infections. Biomedicines.

[15] Berbari E., Mabry T., Tsaras G., Spangehl M., Erwin P.J., Murad M.H., Steckelberg J., Osmon D. (2010). Inflammatory blood laboratory levels as markers of prosthetic joint infection: A systematic review and meta-analysis. J. Bone. Joint. Surg. Am..

[16] Meier M.-P., Bauer I.J., Maheshwari A.K., Husen M., Jäckle K., Hubert J., Hawellek J., Lehmann W., Saul D. (2021). Predicting the expection—CRP and primary hip arthroplasty. J. Clin. Med..

[17] Xu C., Tan L., Kuo F.C., Goswami K., Wang Q., Parvizi J. (2019). Reevaluating current cutoffs for acute periprosthetic joint infection: Current thresholds are insensitive. J. Arthroplast..

[18] Schäfer P., Fink B., Sandow D., Margull A., Berger I., Frommelt L. (2008). Prolonged bacterial culture to identify late periprosthetic joint infection: A promising strategy. Clin. Inf. Dis..

[19] Siekmeier R., Bierlich A., Jaross W. (2001). The white blood cell differential: Three methods compared. Clin. Chem. Lab. Med..

[20] Arroyo M.E., Tabernero M.D., Garcia-Marcos M.A., Orfao A. (2005). Analytic performance of the PENTRA 80 automated blood cell analyser for the evaluation of normal and pathologic WBCs. Am. J. Clin. Pathol..

[21] Danise P., Maconi M., Rovetti A., Avino D., Di Palma A., Gerardo Pirofalo M., Esposito C. (2013). Cell counting of body fluids: Comparison between three automated haematology analysers and the manual microscope method. Int. J. Lab. Hematol..

[22] Krenn V., Otto M., Morawietz L., Hopf T., Jakobs M., Klauser W., Schwantes B., Gehrke T. (2009). Histopathologic diagnostics in endoprosthetics: Periprosthetic neosynovialitis, hypersensitivity reaction, and arthrofibrosis. Orthopäde.

[23] Krenn V., Morawietz L., Perino G., Kienapfel H., Ascherl R., Hassenpflug G.J., Thomsen M., Thomas P., Huber M., Kendoff D. (2014). Revised histopathological consensus classification of joint implant related pathology. Pathol. Res. Pract..

[24] Müller M., Morawietz L., Hasart O., Strube P., Perka C., Tohtz S. (2009). Histopathological diagnosis of periprosthetic joint infection following total hip arthroplasty: Use of a standardized classification system of the periprosthetic interface membrane. Orthopäde.

